# Effect of antioxidant treatment with n-acetylcysteine and swimming on lipid expression of sebaceous glands in diabetic mice

**DOI:** 10.1038/s41598-021-91459-x

**Published:** 2021-06-07

**Authors:** Geovane Ribeiro Santos, Marcelo Rodrigues Cunha, Eduardo José Caldeira, Ewerton Alexandre Galdeano, Raphael Cruz Seabra Prudente, Clóvis Antonio Lopes Pinto

**Affiliations:** 1 Morphology and Basic Pathology Department, Jundiaí Medical School (JMS) Jundiaí, Francisco Telles, 250 - Vila Arens II, 1109, Jundiaí, SP 13202-550 Brazil; 2 Pathology and Cytology Laboratory, Jundiaí Medical School (JMS) Jundiaí, Jundiaí, São Paulo Brazil; 3 Institute of Health Sciences, ICS, of the Paulista University, UNIP (Campus Jundiaí), Jundiaí, São Paulo Brazil; 4 Health Management and Promotion Unit (HMPU), Jundiaí, São Paulo Brazil; 5 Pathological Anatomy Service of Hospital A.C. Camargo, São Paulo, SP Brazil

**Keywords:** Biochemistry, Biological techniques, Cell biology, Chemical biology, Physiology, Biomarkers, Diseases

## Abstract

The sebaceous gland (SG) is involved in different inflammatory, infectious and neoplastic processes of the skin and can be related to specific diseases, e.g., diabetes mellitus. Sometimes, the histological diagnosis requires complementary tests due to the ability of diseases to mimic other tumors. We evaluated the sebaceous gland density in Non-obese diabetic mice to analyze the N-acetylcystein effects and swimming exercise treatment in sebaceous glands healing, using specific staining in histochemistry and immunohistochemistry reactions in the identification of the lipid expression in the sebaceous gland. We investigated the intracytoplasmic lipid expression and analysis of gland density from SG in dorsal skin samples from the Non-obese diabetic (NOD mice) and diabetic animals submitted to antioxidant treatment and physical exercise. For histological analysis of the sebaceous glands, specific staining in histochemistry with sudan black and immunohistochemistry reaction with adipophilin were used in the evaluation. Statistical analysis showed significant proximity between the values of the control group and the diabetic group submitted to the swimming exercise (DS group) and similar values between the untreated diabetic group (UD group) and diabetic group treated with the antioxidant N-acetylcysteine (DNa group), which did not prevent possible differences where p < 0.01. Adipophilin (ADPH) immunohistochemistry permitted more intense lipid staining in SGs, the preservation of the SG in the control group, and a morphological deformed appearance in the UD and DNa groups. However, weak morphological recovery of the SG was observed in the DS-Na group, being more expressive in the DS group. In conclusion, the groups submitted to physical exercises showed better results in the recovery of the analyzed tissue, even being in the physiological conditions caused by spontaneous diabetes.

## Introduction

Diabetes mellitus is a global public health problem that affects millions of people and causes severe pathological alterations in different organism systems, including the integumentary system^1,2^. In the skin, diabetes has been shown to cause cell atrophy and biomembrane disorganization^3–5^, as well as increased fibrillar components of the extracellular matrix, the number of inflammatory cells and some enzymes such as catalase, promoting changes in the morphology and physiology of sebaceous glands^6–10^.

The sebaceous glands (SGs) are involved in infectious and inflammatory processes such as acne, keratosis pilaris, acne rosacea, and some cysts^11,12^, as in the development of neoplasms such as sebaceous adenoma, sebaceoma and carcinoma of the SG, the latter being a rare and aggressive type of cancer^13,14^. The histological diagnosis sometimes requires complementary tests due to the rarity of SG diseases and their capacity to mimic other skin tumors. The diagnosis of these diseases is histologically confirmed by the finding of cells with fat vacuoles in the tumor mass, which are often scarce^14^. Previous studies have reported the challenge in the diagnosis of SG diseases, as well as in their treatment^15–17^.

Different alternatives exist for the treatment of diabetes, such as daily insulin application and glucose monitoring, diet changes, use of therapeutic drugs like N-acetylcysteine (NAC), and regular physical activity^18–20^. NAC is an antioxidant that is able to stimulate the synthesis of reduced glutathione (GSH), the enzyme responsible for the antioxidant defense system. Inside the cell, acetylcysteine is deacetylated to l-cysteine, an amino acid that is essential for the GSH^19,21^. GSH is an important tripeptide for cell integrity that acts as an intracellular defense against exogenous and endogenous oxidant radicals and against cytotoxic substances^19^. Furthermore, GSH induces an increase in CD4 and CD8 T lymphocytes infiltration, promoting the disease’s development. The association of a specific antibody is necessary to reduce CD4 and CD8 T cell infiltration in the pancreas, preventing the destruction of beta cells^21–25^.

Physical activity is an important tool for glycemic control^26,27^. Regular physical activity increases energy expenditure and contributes to weight loss^27^. Exercise-mediated energy expenditure assists in glycemic control since it promotes consumption of glucose stored in blood^26^. In addition to reducing glucose levels, physical exercise improves insulin sensitivity, stimulates calorie burning, and induces the release of hormones such as estrogen^7,8^, cortisol and testosterone, among other benefits^26,27^. This physiological response during the treatment provides the morphological revitalization. Histopathological analyzes are essential to assist in the diagnosis of the lesion, as well as in the evaluation of the tissue after treatment^16,17^.

One method frequently used for the detection of lipids in the pathological diagnosis of inflammatory and neoplastic processes involving SGs is histochemical staining with sudan^3,7,28,29^, characteristic to stain lipids in fresh tissues^30^; however, its use is limited due to the difficulty level of this technique in allowing sebaceous differing and its application to paraffin-embedded materials^30,31^. As a consequence of these limitations, other markers are explored as alternatives, for example, antibodies used to detect the immunohistochemistry expression of lipids^32–34^.

Immunohistochemistry allows the tracking of specific proteins^32–34^. Furthermore, it is superior to protein identification methods based on the demonstration of amino acids since it can detect molecules such as specific tissue antigens^34^. This procedure is therefore important for the diagnosis of inflammatory and infectious diseases, as well as for the identification of prognostic factors for skin lesion treatment^4,34–36^. These markers of lipid expression, can be used in specific clinical situations that alter the histophysiology of this gland, such as sebaceous hyperplasia, folliculitis, steatocystoma, sebaceous adenocarcinoma, sebaceous carcinoma, and skin disorders caused by diabetes mellitus^35,37,38^.

The objective of the study was to evaluate the sebaceous gland density in Non-obese diabetic mice and healthy mice to analysis the effects of the N-acetylcystein and swimming exercise treatment in Sebaceous Glands recuperation. The study also evaluated the efficacy of histochemistry coloring with Sudan Black stain markers to evaluate the sensibility of the immunohistochemistry with Adipophil-in (ADPH) antibody in identification of the lipidic expression in sebaceous gland.

## Materials and methods

### Experimental design

25 female mice (15 weeks-old, weight average = 20 g), obtained from the Multidisciplinary Center for Biological Research of Universidade Estadual de Campinas, being 20 non-obese diabetic (NOD) mice and 5 inbred control BALB/c mice were used on the experiment. The animals were divided into the following groups of five animals each: (1) control group, with healthy BALB/c mice; (2) untreated NOD mice group; (3) NOD mice group submitted to swimming exercise; (4) NOD mice group treated with NAC; (5) NOD mice group submitted to swimming exercise and treated with NAC. This work was supported by the Nucleus of Support to Research and Teaching (NAPED), Faculty of Medicine of Jundiaí, and Research Foundation of the State of São Paulo (FAPESP), protocol number: 2012∕18012-2). The study was approved by the Ethics Committee on Animal Experimentation of the Faculty of Medicine of Jundiai, Brazil (Protocol 351/2011). Experiments were performed in accordance with all relevant guidelines and regulations. The authors followed throughout the experiment to the ARRIVE guidelines based on the principles of NC3Rs.

### Intervention

After the validation of hyperglycemic condition, the mice from the DS group were submitted to swimming exercises with an average duration of 30 min, five times a week for 21 days. The description of the swimming method was adjusted, we added the water temperature and the depth of the swimming pool used. The training was done in a similar method to the previously developed mice protocol, where proper aerobic and cardio physical training intensity, frequency and duration were carefully studied^19^. Mice from the DNa group received a daily intraperitoneal dose of 50 mg/kg NAC and a weekly intravenous dose of 25 µl anti-CD4 and anti-CD8 antibodies, to block the negative effects of NAC (mainly the lymphocyte stimulation) for 21 days. Previous group studies showed a reduction in lymphocyte activity in pancreatic beta cells submitted to treatment with anti-CD4 and anti-CD8, in the same interventions described in this work^23,24^. In animals from the DS-Na group, both treatments from the DS and DNa groups were applied.

Urinary glucose (mg/dL) levels were monitored daily for all subjects. Urine was collected daily by manual compression of the bladder, discarding the first drop. Next, the concentrated urine was analyzed with a reagent strip (MultistiK 10 SG Bayer) and the reference table provided by the manufacturer was used for glucose levels measurement.

The animals were classified as diabetic when glucose levels were higher than 300 mg/dL. To confirm and guarantee the reliability of the values indicating the hyperglycemic state of the animals, blood glucose levels were measured weekly with a Roche Accu-Chek Performa glucose meter and reagent strips (New York, USA).

### Skin samples collection

After the experimental period, the subjects were anesthetized with ketamine (Ketalar, Parke-Davis) and xylazine (Rompun, Bayer Animal Health) in doses of 0.02 g/ml for skin tissue samples removal. In the study, we conducted a pilot group to evaluate the areas where samples were collected, through the routine HE methodology, which presented us with the dorsal area near the skull region, with a good amount of SGs, with good cell volume. After this process, the animals were sacrificed with an anesthetic overdose according to ethical guidelines (Ethical Process Number: 351\2011).

### Histological techniques

#### Tissue fixation

The samples collected were divided into two fragments submitted to different tissue fixation techniques. Both fragments were stained with hematoxylin–eosin, but one was submitted to histochemistry and the other to immunohistochemistry for lipid expression analysis in SGs.

The first fragment was prefixed in phosphate-buffered saline solution, then embedded in Tissue Tek OCT (optimal cutting temperature) compound, frozen in liquid nitrogen, and stored in a biofreezer at – 80 °C for subsequent histochemical analysis. The second fragment was fixed in 10% formalin buffered with anhydrous dibasic and monobasic sodium phosphate. Next, the fragment was submitted to routine processing for dehydration and embedding in paraffin. Histological sections (4 µm) were cut for immunohistochemical analysis.

### Histochemical staining

The samples were cut into 8-µm thick sections with a cryostat for staining with Sudan Black. For this purpose, the sections were rinsed in 70% alcohol, immersed in a saturated solution of Sudan Black for 2 h, and again rinsed in 70% alcohol to remove excess dye. The sections were washed in distilled water and dried at room temperature. The slides were counterstained with panoptic stain, washed in deionized water, and coverslipped.

### Immunohistochemistry reaction

For immunohistochemistry, the slides were immersed in an antigen retrieval solution consisting of EDTA/Tris, pH 9.0, at 125 °C in an electric pressure cooker managed by a REPTIDE microprocessor (Celerus). Nonspecific binding of the antibodies was blocked by incubation of the slide with hydrogen peroxide. Next, the slides were incubated with the primary ADPH antibody (clone AA5-27, LifeSpan) diluted 1/100. To amplify the antigen–antibody binding, two other antibodies were applied to the slides, post-primary (Novolink polymer Novocastra) and the polymer (Novolink polymer Novocastra). The reaction was developed with diaminobenzidine (DAB) as a chromogen (Liquid DAB + Substrate Chromogen System Kit, DakoCytomation, Carpinteria, CA, USA).

### Microscopic analysis

The slides were analyzed and photographed under an Olympus BX-41 light microscope (Olympus, Japan), equipped with an image capture system. All observations were made with achromatic objectives of the 20×, 40× and 60× planes. These slides were also used for quantification of three-dimensional tissue allowing to analyze the density of SGs. The images were analyzed using the Sensus digital imaging software of the Olympus cell. The area and perimeter of five SGs of each staining method were measured per animal.

### Stereological and statistical analysis

The SG area (µm^2^) and perimeter (µm) were calculated in the groups studied by ADPH immunohistochemistry, since this was the best marker of this tissue. The results were analyzed by analysis of variance (ANOVA) using the BioEstat 5.3 software. When a difference was observed between the groups studied, the Tukey test was applied at a minimum significance level of 5% (p < 0.05).

### Ethics approval

The study was approved by the Ethics Committee on Animal Experimentation of Jundiaí Medical School, Jundiaí, Brazil (Protocol 351/2011). Experiments were performed in accordance with relevant guidelines and regulations.

### Consent for publication

All authors have read and agreed with the terms for publication.

## Results

### Glycaemic levels

Diabetic subjects presented glucose levels average of 505 mg/dl. The average levels of control animals were 118 mg/dl.

### Comparison of sebaceous gland morphology between groups

**Figure 1:**
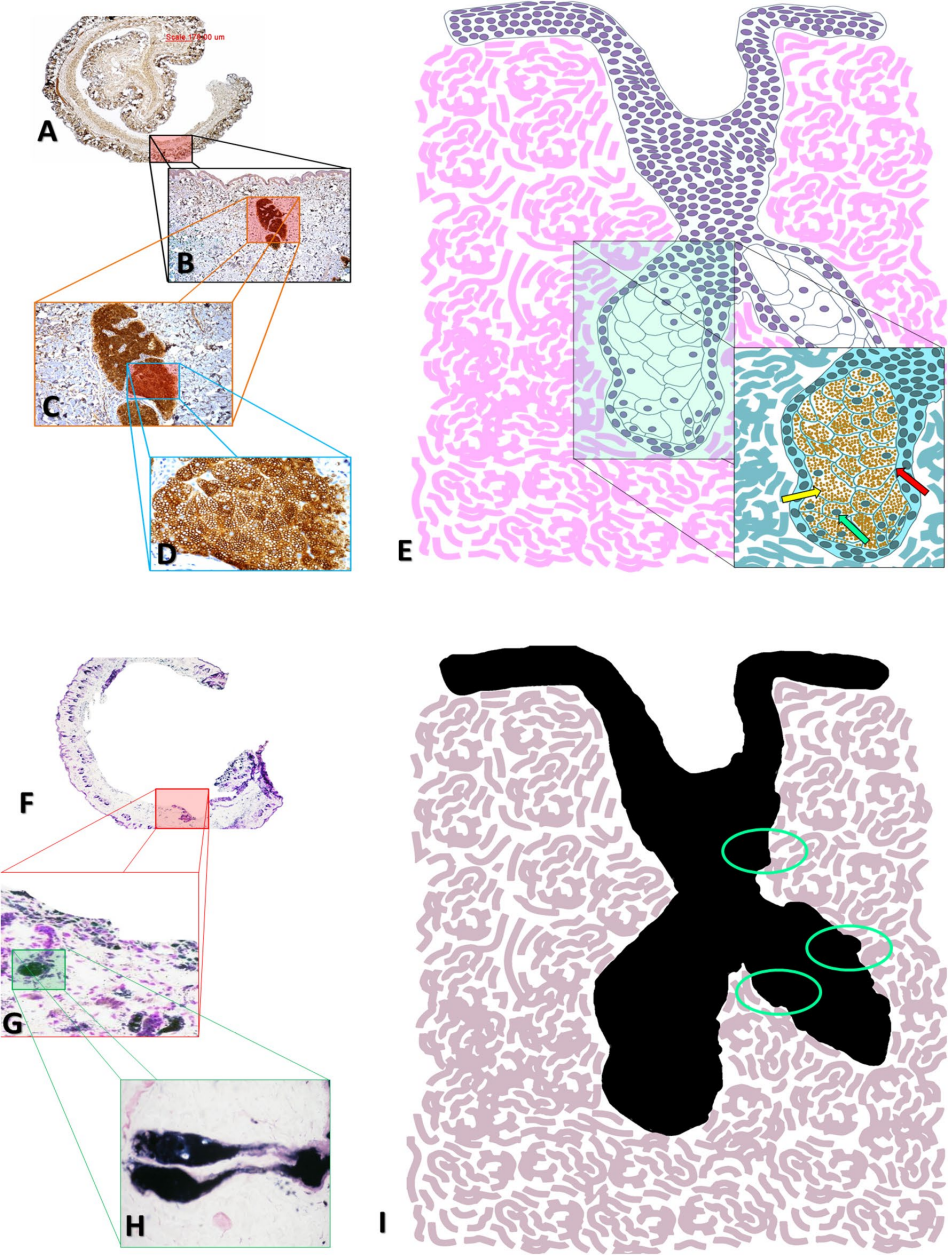
In (**A**–**D**) photomicrographs of mice tissues sections, evaluated in different enlargement in the microscopy, allowed us to observe how much the immunohistochemical technique by ADPH becomes essential, due to its sensitivity, to allow sebaceous differentiation in paraffin-embedded material, as demonstrated in the scheme represented in (**E**). In (**F**–**H**), we have photomicrographs of the sections of the mice’s frozen tissue stained with Sudan Black. Note the presence of black spots, provided by the labeling of the many lipids present in fresh tissue. This technique, when evaluated in a different objective, does not allow the sebaceous differentiation, as demonstrated in the scheme in (**I**).

**Figure 2:**
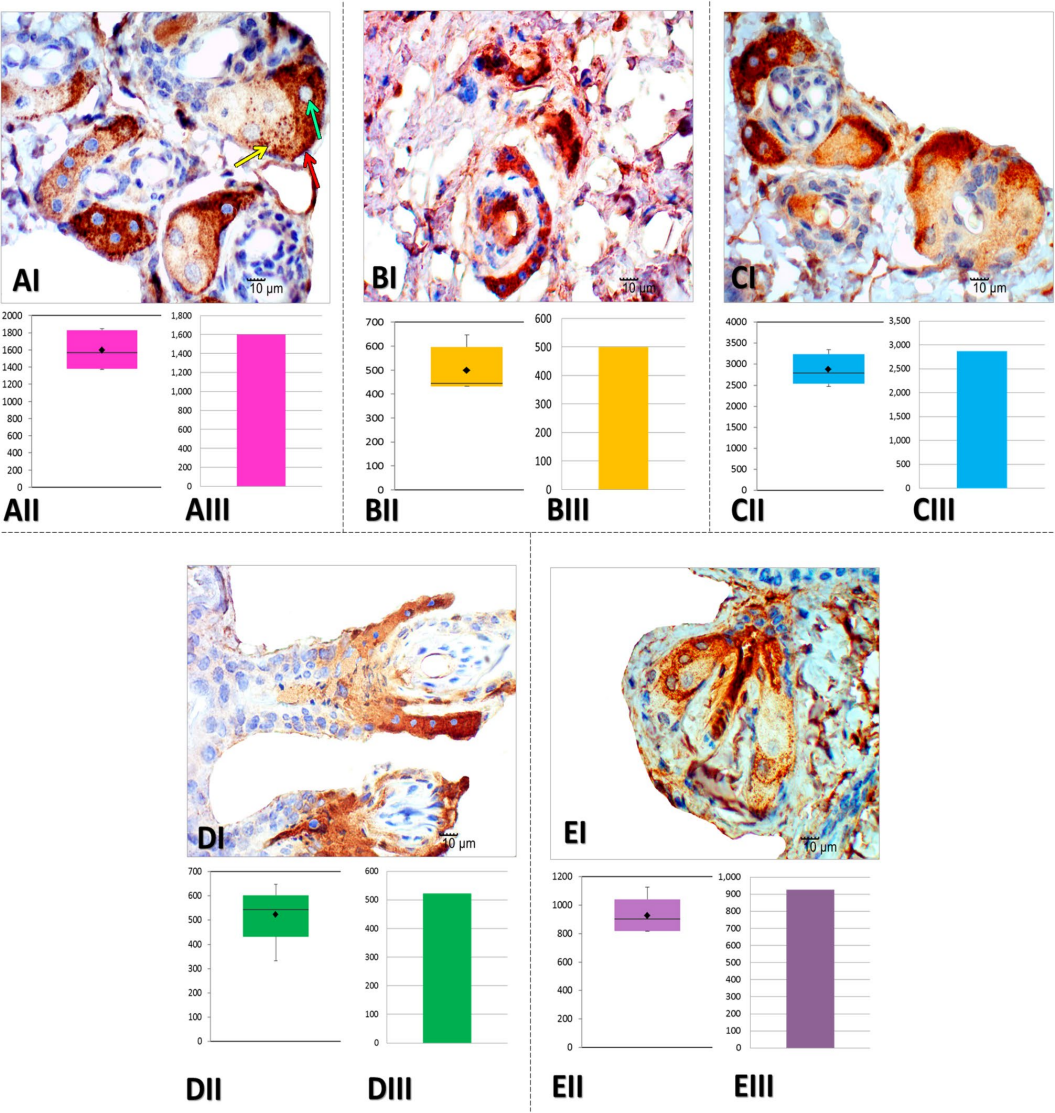
Photomicrographs (×600) of paraffinized tissue sections of mice analyzed by ADPH immunohistochemistry. (**AI**) Notice the fine staining of fat and intracytoplasmic lipid expression in sebaceous cells (yellow arrow). This technique allows us to identify cellular limits (red arrow) and the presence of nuclei (green arrow), as well as expressive differences in the sebaceous glands’ size. (**AI**) Control group. (**BI**) UD group. (**CI**) DS group. (**DI**) DNa group. (**EI**) DS-Na group. (**AII**,**BII**,**CII**,**DII**,**EII**): sebaceous gland area (µm^2^) box plot evaluated in control group, UD group, DS group, DNa group and DS-Na group, respectively. (**AIII**,**BIII**,**CIII**,**DIII**,**EIII**) Average of the sebaceous gland area (µm^2^) evaluated in the control group, UD group, DS group, DNa group and DS-Na group, respectively.

**Figure 3:**
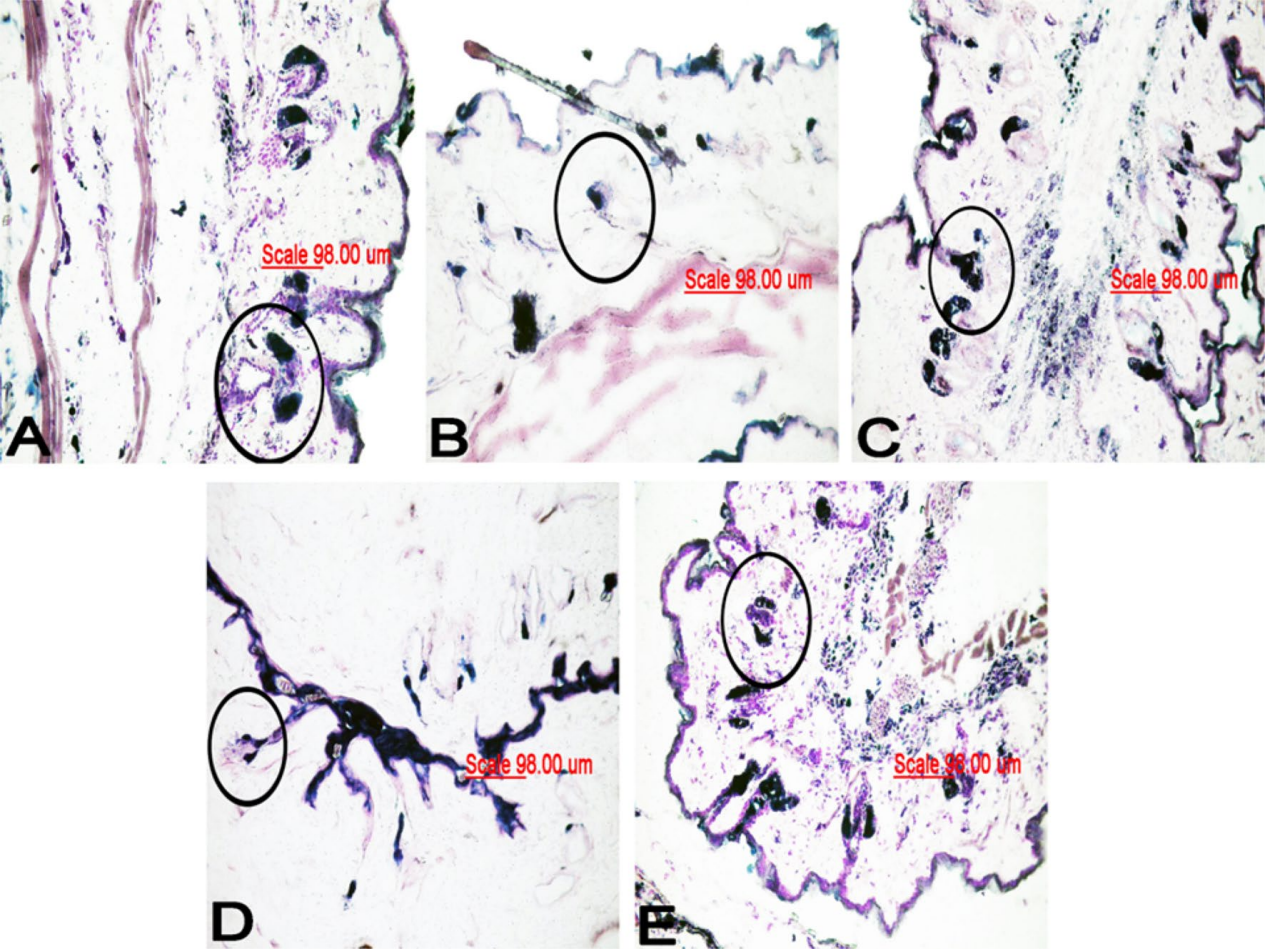
Photomicrographs (×200) of frozen mice tissue sections stained with Sudan Black. Note the presence of a fat spot and intracytoplasmic lipid expression in sebaceous cells. The technique permitted visualization of differences in the size of the sebaceous glands (black circle). (**A**) Control group. (**B**) UD group. (**C**) DS group. (**D**) DNa group. (**E**) DS-Na group.

In the control group, normal SGs with an alveolar shape were noted in the dermis. The glands contained voluminous sebaceous cells with well-defined nuclei and intracytoplasmic vesicles. This SG morphology was characterized by ADPH immunohistochemistry (Figs. 1 and 2AI). Sudan staining only allowed lipid staining without defined sorting of the intracellular content (Figs. 1 and 3A).

In the UD group, immunohistochemistry permitted the observation of fragmented and deformed SGs with an irregular orientation in the dermis. Sebaceous cells’ nuclei identification was challenging. However, intracytoplasmic vesicles staining was possible (Fig. 2BI). The Sudan Black staining allowed the SGs localization, but cellular content differentiation is not possible (Fig. 3B).

Immunohistochemistry (Fig. 2CI) and Sudan staining (Fig. 3C) revealed the recovery of the SG morphology in the DS group, resembling that of the control group, with the observation of marked glandular volume. Opposite results were obtained for the DNa (Figs. 2DI and 3D) and DS-Na (Figs. 2EI and 3E) groups, in which the glands exhibited a deformed shape similar to the UD group. This demonstrates that the group of diabetic mice treated with the antioxidant NAC did not express sebaceous gland recovery.

### Comparison of sebaceous gland morphology between the histological techniques used

**Figure 4:**
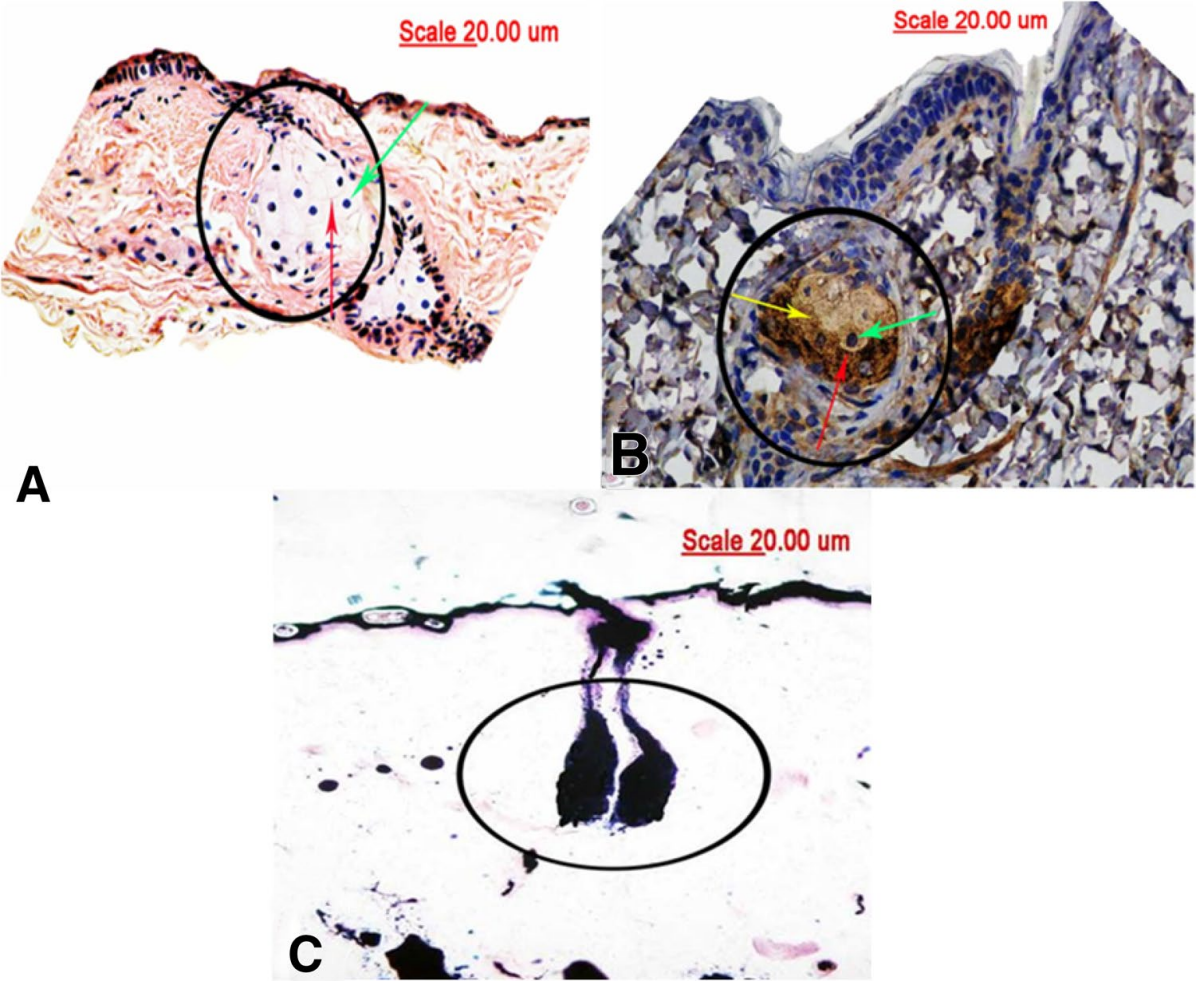
Photomicrographs (×400) of paraffinized tissue sections of control mice allowing, in (**A**), the cellular limits identification (red arrow) and the presence of nuclei (green arrow) by HE staining. In (**B**), notice the good expression of intracytoplasmic lipids in sebaceous cells (yellow arrow), allowing the identification of cellular limits (red arrow) and the presence of nuclei (green arrow) by ADPH immunohistochemistry. (**C**) Shows frozen tissue sections of the control group mice stained with Sudan Black. Notice the presence of tissue fat spots and lipid expression in sebaceous glands (black circle).

ADPH immunohistochemistry, which allowed the use of tissue sections obtained from paraffin-embedded samples used for HE staining, showed a good expression of intracytoplasmic lipids through brown staining of the membranes of intracytoplasmic vesicles, and the nuclei of sebaceous cells could be clearly identified. Histochemical staining with Sudan Black showed strong marking (Fig. 4).

### Stereological and statistical analysis

Sebaceous gland area average (μm^2^) was 1598.494 for the control group (Fig. 2AII,III), 500,324 group UD (Fig. 2BII,III), 2868,100 group DS (Fig. 2CII,III), 522,782 group DNa (Fig. 2DII,III) and 925,71 DS-Na group (Fig. 2EII,III). Statistical analysis showed proximity between the values of the control and DS groups and between the UD and DNa groups, which did not prevent possible differences where p < 0.01.

## Discussion

When analyzing the skin samples’ morphology from the control group, we observed that SGs had an alveolar shape and contained round cells and cytoplasmic lipid accumulation. In the UD group, cellular atrophy of the SG was observed. The deleterious effects of diabetes on SGs are related to hyperglycemia, which induces cellular abnormalities through different metabolic pathways such as non-enzymatic protein glycosylation, activation of aldose reductase and diacylglycerol phosphatidyl kinase C, and reduced hydration through the inhibition of insulin signal transduction^1,3,4^.

Tissue recovery of the SG was observed in animals submitted to swimming exercise (DS group), with the glands exhibiting characteristics similar to those seen in the control group and being even more voluminous in some cases. Stereology confirmed this result, with the observation of similar SG areas in the control and DS groups. This finding can be corroborated by studies that explain the fact that physical exercise promotes blood circulation and improves the body organs’ oxygenation, including the skin^26,27^. Studies have shown that exercise induces a reduction in serum glucose levels by using energy sources that tend to release hormones such as estrogen, cortisol and testosterone^19,26,27^. Insulin, in conjunction with estrogen, can promote the management of glucose levels and the restructuring of tissues damaged by this disease^3,9,27^.

No morphological recovery of SGs was observed in the DNa group, which showed features similar to those seen in the UD group. The same was observed in the stereological analysis of gland volume. These findings agree with the results of other studies demonstrating the lack of NAC treatment efficacy for tissue recovery in diabetic animals^21^. In the DS-Na group, recovery of SG morphology and volume was observed in the animals, but was still less pronounced than that seen in the control and DS groups. These findings suggest that physical exercise promotes the recovery of SGs, but this process is slow due to the antagonistic action of NAC.

NAC is a potent antioxidant due to its capacity to stimulate the synthesis of GSH^22^, the enzyme responsible for the antioxidant defense system^25^. However, NAC can increase the infiltration of CD4 and CD8 T lymphocytes in the pancreas, causing the destruction of beta cells and consequently aggravating the effects of diabetes on tissues^23,24^.

The morphological features in the SGs of diabetic and treated groups were better demonstrated by ADPH immunohistochemistry compared to Sudan staining^39,40^, the former showing an advantage in distinguishing diagnostics contribution^40^. According to Heid^35^ and Muthusamy^33^, ADPH is a specific marker of lipid accumulation which stains the membranes of intracytoplasmic lipid vesicles in a brown color^32–34^.

It is well established that the histochemical staining by Sudan is ineffective in paraffin-embedded samples used for HE staining^3,7,28^. Consequently, Santos et al.^3^ corroborate showing good lipid marking in histochemistry and the impossibility of differentiation of sebaceous cells with Sudan^3^.

## Conclusions

This study shows that exercise is found to be a suitable intervention, promoting quality of life and contributing to tissue recovery during diabetes treatment. On the other hand, the NAC was an ineffective treatment option for the recovery of SGs from diabetic mice. Experiments show that immunohistochemistry is a suitable method for this type of evaluation and indicates that the ADPH marker could be part of a routine immunohistochemical panel due to its efficacy in intracytoplasmic lipidic expression investigations.
